# Interferon-Tau in Maternal Peripheral Blood and Its Relationship with Progesterone and Pregnancy-Associated Glycoproteins in the Early Phases of Gestation in Water Buffalo

**DOI:** 10.3390/ani14111658

**Published:** 2024-05-31

**Authors:** Olimpia Barbato, Laura Menchetti, Anna Beatrice Casano, Giovanni Ricci, Giovanna De Matteis, Stella Agradi, Giulio Curone, Gabriele Brecchia, Emilia Larisa Achihaei, Vittoria Lucia Barile

**Affiliations:** 1 Department of Veterinary Medicine, University of Perugia, Via San Costanzo 6, 06100 Perugia, Italy; olimpia.barbato@unipg.it (O.B.); giovanni.ricci@unipg.it (G.R.); larisa.achi@gmail.com (E.L.A.); 2 School of Bioscience and Veterinary Medicine, University of Camerino, Via Circonvallazione 93/95, 62024 Matelica, Italy; stella.agradi@unicam.it; 3 Istituto Zooprofilattico Sperimentale della Lombardia e dell’Emilia Romagna “Bruno Ubertini”, Via Bianchi 9, 25124 Brescia, Italy; annabeatrice.casano@libero.it; 4 Research Centre for Animal Production and Aquaculture, Consiglio per la Ricerca in Agricoltura e l’Analisi dell’Economia Agraria (CREA), Via Salaria 31, 00015 Monterotondo, Italy; giovanna.dematteis@crea.gov.it (G.D.M.); vittorialucia.barile@crea.gov.it (V.L.B.); 5 Department of Veterinary Medicine and Animal Sciences, University of Milano, Via dell’Università 6, 26900 Lodi, Italy; giulio.curone@unimi.it (G.C.); gabriele.brecchia@unimi.it (G.B.)

**Keywords:** interferon tau, progesterone, pregnancy-associated glycoproteins, pregnancy, buffalo

## Abstract

**Simple Summary:**

Understanding the intimate mechanisms linked to embryo survival in livestock represents an important step in reducing the incidence of early embryonic losses, which cause a significant delay in the calving–conception period and, as a consequence, a delay in production processes and economic income. The aim of this study was to investigate the interferon tau (IFNt) concentration in the peripheral maternal blood during the early phase of pregnancy in buffalo cows and improve the knowledge on the physiological importance of circulating IFNt, evaluating the possible interaction with pregnancy-associated glycoproteins (PAGs) and progesterone (P4). This is the first report that shows the possibility of detecting the maternal circulating IFNt in buffalo cows during the early period of gestation, showing different concentrations among pregnant and non-pregnant animals and those that experience embryo mortality. Close associations among IFNt, PAGs and P4 during the sensitive period in which the conceptus must manifest its presence demonstrate that all three molecules work together for fetal–placental well-being and pregnancy support. Unfortunately, the great individual variability in circulating IFNt makes this analysis unsuitable for early pregnancy diagnosis.

**Abstract:**

The aim of this study was to investigate the interferon tau (IFNt) concentration in the peripheral maternal blood during the early phase of pregnancy in buffalo cows and improve the knowledge on the physiological importance of circulating IFNt, evaluating the possible interaction with pregnancy-associated glycoproteins (PAGs) and progesterone (P4). Blood samples were taken from buffalo cows on day 0 (day of AI), 7, 14, 18, 28, and 40 post insemination for the IFNt, PAG, and P4 analysis and to determine the IFNt mRNA expression. The animals were categorized ex post into Pregnant, Non-pregnant and Embryo mortality groups. The interferon value was influenced by group (*p* = 0.003), being always higher in pregnant buffalo cows than in non-pregnant ones, while the embryo mortality group showed intermediate values between those for pregnant and non-pregnant animals. The mRNA expression of IFNt was not influenced by groups or any time points. The regression analysis that included IFNt as the independent variable showed that PAGs, from day 18 (*p* < 0.01), and P4, from day 28 (*p* < 0.05), were positively associated with IFNt values. The close associations among IFNt, PAGs and P4 demonstrate that all three molecules work together for fetal–placental well-being and pregnancy support. Unfortunately, the great individual variability in circulating IFNt makes this analysis unsuitable for early pregnancy diagnosis.

## 1. Introduction

In ruminants, as in all mammals, pregnancy success depends on a series of complex biological processes and several crucial points; among these, adequate communication between mother and embryo is necessary to create a uterine environment capable of ensuring the implantation and survival of the embryo [1]. Understanding the intimate mechanisms linked to embryo survival represents an important step in reducing the incidence of early embryonic losses, which cause a significant extension of the calving to conception interval and, as a consequence, a delay in the onset of subsequent lactation, resulting in a negative economic impact [2].

One of the first molecules involved in the complex mechanism of maternal recognition of pregnancy (MRP) is the interferon-tau (IFNt); it belongs to the Type I cytokine family and has antiviral, antiproliferative and immunomodulatory biological effects [3]. Type I interferons with a high degree of structural homology include the interferons -alpha, -beta, -delta, -epsilon, -kappa, -tau and -omega. Among these, IFNt is the only one involved in the pregnancy recognition signal [4]. In ruminants, it is secreted by the mononucleate trophoblastic cells during the peri-implantation period; it increases with the elongation of the conceptus and then stops when trophectoderm cells appose the luminal endometrial epithelium [4,5,6]. In buffalo, Saugandhika et al. [7] reported that it is secreted by the trophectoderm at around days 16–25 of gestation. The importance of IFNt in MRP is that it prevents luteolysis by inhibiting prostaglandin F2α (PGF2α) release, resulting in the maintenance of the corpus luteum (CL) function [8]. Moreover, IFNt is involved in regulating immunocyte populations and the products of lymphocytes in the endometrium [9]. Another role of conceptus-derived IFNt is to regulate the transcripts of interferon-stimulated genes (ISGs) controlling uterine receptivity and conceptus elongation [10,11,12,13].

In ruminant species, other molecules synthesized by the trophoblast implicated in the complex mechanism of maintenance of pregnancy and fetal–placental well-being are the pregnancy-associated glycoproteins (PAGs) [14,15,16]. These proteins have a possible immunomodulatory role necessary for the establishment and the maintenance of the maternal–fetal unit histocompatibility [17,18]. In vitro studies reported that PAGs induce the release of prostaglandin E2 (PGE2) and P4 from luteal cells, and PGE2 from endometrial cells, giving them a possible luteotropic role [19,20]. PAGs are released into the maternal circulation at the time of implantation, and their detection in the blood has become a useful tool for monitoring pregnancy and fetal–placental unit viability. They, therefore, represent a reliable method for quickly revealing the presence of placental alterations and embryonic losses [15,21,22]. In buffalo, after 25 days of gestation, PAG concentrations enable discrimination between animals that are pregnant and non-pregnant and those that have experienced embryonic mortality [23,24,25].

On the other hand, maternal-secreted hormones are also pivotal to the maintenance of pregnancy. Specifically, P4 secreted by CL during gestation has been the subject of several reviews in ruminants [26,27,28,29,30]. This hormone plays a key role in conceptus development, uterine receptivity, and the establishment and maintenance of pregnancy through its effects on oocyte quality and the uterine endometrium. Numerous studies have demonstrated that elevated concentrations of circulating P4 in the period immediately following conception are associated with advancement in conceptus elongation, increased IFNt production, and higher pregnancy rates; conversely, low P4 concentrations are associated with reductions in conceptus growth and elongation, decreased IFNt production, and lower pregnancy rates [29,30,31]. Therefore, there is a close relationship between P4 and IFNt in the delicate phase of the peri-implantation period.

To the best of our current knowledge, there are no studies investigating the presence of IFNt in the maternal peripheral circulation during early pregnancy in ruminants, aside from a small number of reports on bovines [32,33]. It is commonly known that a small amount IFNt is released into the blood during the period of MRP, while a large amount is retained inside the uterus [34]. 

Therefore, the aim of this study was to investigate IFNt concentrations in the peripheral maternal blood during the early phase of pregnancy in buffalo cows and improve our knowledge about the physiological importance of circulating IFNt, while also evaluating the its possible interaction with PAGs and P4.

## 2. Materials and Methods

### 2.1. Animals and Experimental Design

The study was carried out at the CREA Animal Production and Aquaculture experimental farm, in Monterotondo, Rome, Italy. The experimental procedures were assessed and approved by the CREA Committee of Ethics in Animal Research (Protocol N.0081676-02/11/2020).

In this study, 40 pluriparous Italian Mediterranean buffalo cows (5–9 years old; average body weight of 734 ± 55.44 kg) undergoing a synchronization and artificial insemination (AI) program were enrolled and grouped ex post as Pregnant (n = 18), Non-pregnant (n = 16) and Embryo mortality (n = 6) groups, based on the diagnostic criteria described below in Section 2.2.

The buffaloes were kept in a loose-housing system, fed ad libitum once a day with a total mixed ration based on sorghum silage, hay and concentrate and milked twice a day in a milking parlor.

The buffalo cows were synchronized with a progesterone-releasing intravaginal device (PRID^®^, Sanofi, France) associated with PMSG, PGF2α and GnRH, as reported by Barile et al. [35]. The cows were artificially inseminated using frozen–thawed semen 72 h after PRID^®^ removal. Blood was collected from the jugular vein in 10 mL EDTA tubes and immediately processed for peripheral blood mononuclear cells (PBMCs) isolation, as described below in Section 2.6. Plasma was separated by centrifugation at 2700× *g* for 10 min and then stored at −20 °C until assayed.

For the determination of P4 and PAG, samples were collected on days 0, 14, 19, 28 and 40 post AI. For the determination of IFNt and its mRNA expression, samples were collected starting from day 14 post AI.

### 2.2. Pregnancy Diagnosis

Pregnancy was diagnosed using either conventional transrectal ultrasonography on day 30 post AI or PAG determination.

Using transrectal ultrasonography, the buffaloes were considered pregnant if an embryonic vesicle and embryo proper with a beating heart were detected, while in the absence of these conditions, the buffaloes were considered as non-pregnant. Embryo mortality was diagnosed when a vesicle without an embryo proper and/or an embryonic heart beat were not visible [21].

PAG determination was employed as an adjunct to conventional ultrasonography to diagnose pregnant and non-pregnant animals and monitor the pregnant animals throughout the sampling period. Based on PAG plasma levels (cut-off value: 1 ng/mL) [21], the buffaloes were considered non-pregnant when concentrations remained close to zero throughout sampling and pregnant when the concentrations were >1 ng/mL at d 28 and d 40 post AI. For animals in which the PAG concentration was found to be close to the cut-off between 14 and 28, and then found to be below 0.2 ng/mL on day 40, embryo mortality was deemed to have occurred. PAG concentrations allowed better discrimination of pregnant buffaloes from those that experienced embryonic mortality; thus, PAG concentrations were used to differentiate ex post the groups: Pregnant, Non-pregnant, and Embryo mortality. 

Pregnancy status was confirmed on day 60 post AI by rectal palpation.

### 2.3. P4 Radioimmunoassay

Plasma P4 was analyzed by the RIA method as counted in a gamma counter (Packard Cobra II Auto Gamma; PerkinElmer, Shelton, CT, USA) and determined using commercially available kits according to the manufacturers’ instructions (PROG-RIA-CT, KIP 1458, DIAsource ImmunoAssays S.AS, Lovain-la-Neuve, Belgium), which can be utilised for analysis in this species [36]. The sensitivity was 0.05 ng/mL, while the range of detection was between 0.12 and 36.0 ng/mL, and the inter- and intra-assay coefficients of variation for the progesterone assay were 5.0 and 8.7%, respectively.

### 2.4. PAG Radioimmunoassay

RIA-860, as previously described by Barbato et al. [24,37], was used to determine PAG concentration. Pure boPAG_67kDa_ preparation was used as the standard and tracer. Iodination (Na-I^125^, Amersham Pharmacia Biotech, Uppsala, Sweden) was carried out according to the Chloramine-T method previously described by Greenwood et al. [38]. The samples were assayed in a preincubated system in which the standard curve ranged from 0.2 to 25 ng/mL. The minimum detection limit (MDL), calculated as the mean concentration minus twice the standard deviation (mean-2 SD) of 20 duplicates of the zero (B0) standard, was 0.1 ng/mL. The intra- and inter-assay coefficients were 2.5% and 7.5%, respectively.

### 2.5. IFNt Quantitative Sandwich Enzyme Immunoassay 

IFNt in the plasma was determined using the “Bovine IFNt ELISA Kit” (Catalog Number. CSB-E16948B, Cusabio Biotech Co., Ltd., Houston, TX, USA). The sensitivity of the assay was less than 1.56 pg/mL with a detection range from 6.25 to 400 pg/mL. The minimum detection limit (MDL), calculated as the mean concentration minus twice the standard deviation (mean-2 SD) of 20 duplicates of the zero (B0) standard, was 0.3 ng/mL. The intra- and inter-assay coefficients were 2.3% and 7.0%, respectively.

### 2.6. PBMCs Purification, RNA Isolation and RT-qPCR

The PBMCs isolation and the protocols for total RNA extraction, quality assessment and RNA concentration were performed as previously described [13]. Briefly, total RNA was extracted from the PBMCs and lysed in 1.0 mL TRIzol™ Reagent (Invitrogen, Life Technologies, Carlsbad, CA, USA) according to the manufacturer’s protocol. The concentration and quality of RNA were determined by measuring the absorbance and ratio at 260 nm and 280 nm wavelengths. Total RNA (1 μg) was reverse-transcribed using the High-Capacity cDNA Reverse Transcription Kit (Applied Biosystem, Foster City, CA, USA), according to the manufacturer’s instructions. The cDNA obtained from each sample was used as a template for RT-qPCR. The species–specific primer pairs for IFNt and beta-actin (ACTB) were designed by Primer Express Software v5.0 (Applied Biosystems, Foster City, CA, USA) using the buffalo sequences on the NCBI database, and the primer pairs specificity was checked using NCBI Primer-BLAST software (https://www.ncbi.nlm.nih.gov/tools/primer-blast/) (accessed on 1 October 2022), as previously reported [13]. Relative quantification of IFNt transcript was carried out following the MIQE guidelines.

The amplification reaction was conducted in duplicate in a 25 μL mixture containing each sample, 2X Power Sybr Green PCR Master Mix (Applied Biosystem, Foster City, CA, USA) and primers at 500 nM each (0.5 μL of 10 μM solution). A duplicate no-template control (NTC) was also included. RT-qPCR was carried out using the 7000 Sequence Detection System (Applied Biosystem, Foster City, CA, USA) under the following thermal cycle conditions: 10 min at 95 °C followed by 40 cycles of 15 s at 95 °C and 1 min at 60 °C.

Quantification was determined after applying an algorithm to the data analyzed by the 7000 Sequence Detection System software v1.2.3 (Applied Biosystem, Foster City, CA, USA). The amplification efficiency was determined using the slope of the standard curve: Efficiency = 10−1/slope −1. Only PCR reactions with PCR efficiencies > 95% were included in the subsequent analyses. In detail, the amplification efficiency for the IFNt was E = 112.7%, and finally for ACTB, the amplification efficiency was 98.80%.

The expression level of IFNt was normalised using the beta-actin (ACTB) as reference gene levels (mean) of the same sample and run. The relative expression of the target gene was calculated using the 2^−ΔCt^ [39]. The obtained value was multiplied by 10,000 in order to obtain the test Arbitrary Units (AU).

### 2.7. Statistical Analysis

Diagnostic graphs as well as Kolmogorov–Smirnov and Levene’s tests were used to check assumptions and identify outliers. Extreme outliers (more extreme than Q1 − 3 * IQR or Q3 + 3 * IQR) were eliminated. We decided to exclude two animals (ID 4722 and ID 4987) because they showed outliers at all time points. The PAG and IFNt concentrations were Log(x + 1) transformed for the analyses, but raw values were presented as means and standard errors. Changes in PAG, P4, and IFNt values in the three groups over time were analyzed using the Linear Mixed model, in which animals and time points were included as the subject and repeated factors with an “Exchangeable” covariance structure, respectively. Sidak adjustment was used for carrying out multiple comparisons. The models evaluated the main effects of time (i.e., day after AI, 5 levels: 0, 14, 18, 28, and 40 days post AI), outcome (3 levels: Pregnant, Non-pregnant, and Embryo mortality), and their interaction. Moreover, hypothesizing that IFNt may influence PAG and P4 concentrations, univariable linear regressions stratified for each time point were performed including IFNt as an independent variable. In addition to the b coefficient with its stand error and the associated *p*-value, the coefficient of determination R2 was also reported as a result.

Moreover, the biological variations in the parameters were investigated [40,41]. Intra-individual (CVintra) and inter-individual (CVinter) variability were calculated as CV (%) = 1 SD/mean × 100%. The CVintra was estimated from repeated measurements in the same animal, whereas the overall means of the entire sample or group were used to estimate the CVinter. The index of individuality (II) was calculated as CVintra/CVinter. When the index of individuality is low for a given analyte (CVintra < CVinter), that analyte is said to have marked individuality [42,43].

Finally, the correlation between IFNt concentrations and gene expression was evaluated using Spearman’s coefficient (ρ). The correlation was considered poor if ρ < |0.3|, medium if |0.3| ≤ ρ < |0.5|, and large if ρ ≥ |0.5| [44].

The statistical analyses were performed with SPSS (Statistical Package for the Social Sciences) 25.0 software (SPSS Inc. Chicago, IL, USA). A *p*-value ≤ 0.05 was considered to be statistically significant.

## 3. Results

Out of 40 animals enrolled in the study, two animals were excluded from the results (one from the Pregnant group and one from the Non-pregnant group) because they showed outliers at all time points. 

**Figure 1:**
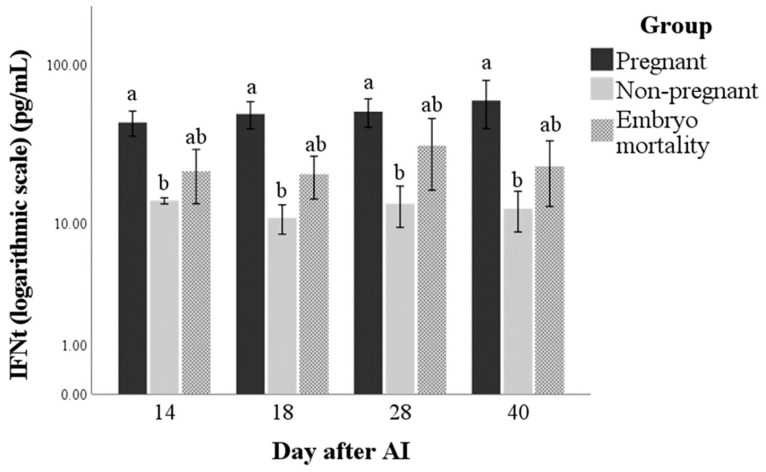
Interferon-tau (IFNt) concentrations in pregnant and non-pregnant buffalo cows and buffalo cows that experienced embryo mortality after artificial insemination (AI). Values are means ± standard error. Bars with different lowercase letters within each time are significantly different at *p* < 0.05.

The IFNt immunoassay utilised in this study was able to detect this protein in maternal circulation in buffalo cows. Interferon-tau was only influenced by group (*p* = 0.003). The values were always higher in the Pregnant group than in the Non-pregnant one (*p* < 0.01), while the Embryo mortality group showed intermediate values between the Pregnant and Non-pregnant groups (Figure 1). The marginal means (±standard error) of the groups in the observation period were 50.9 ± 6.2, 12.5 ± 1.5, and 24.1 ± 4.9 pg/mL for Pregnant, Non-pregnant and Embryo mortality groups, respectively.

**Figure 2:**
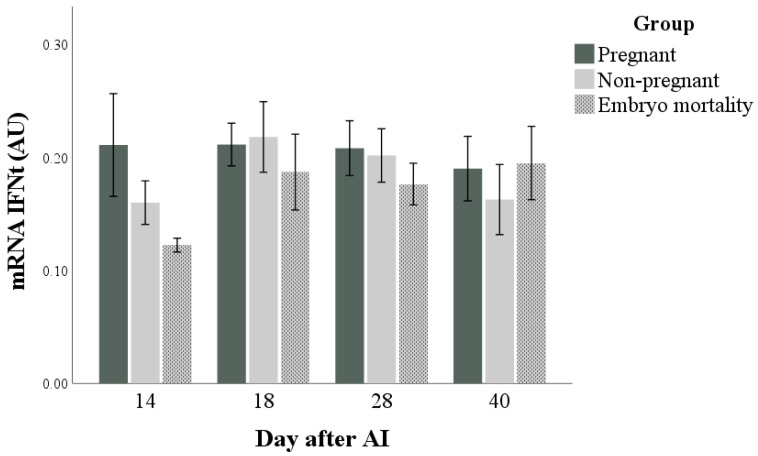
Interferon-tau (IFNt) expression in pregnant and non-pregnant buffalo cows and buffalo cows that experienced embryo mortality after artificial insemination (AI). Values are means ± standard error.

The mRNA expression of IFNt was not influenced by any factor (Figure 2) and did not correlate with IFNt concentrations at any time point (ρ = −0.402, ρ = 0.137, ρ = −0.080, and ρ = 0.068 at days 14, 18, 28, and 40 after artificial insemination; for all: *p* > 0.05). The marginal means (±standard error) of the groups in the observation period were 0.2 ± 0.0, 0.2 ± 0.0, and 0.2 ± 0.0 AU for Pregnant, Non-pregnant, and Embryo mortality groups, respectively.

**Figure 3:**
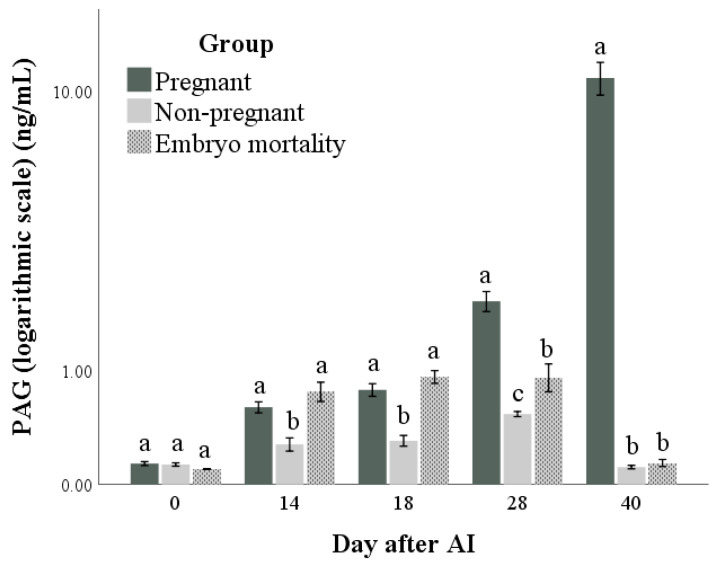
Pregnancy-associated glycoprotein (PAG) concentrations in pregnant and non-pregnant buffalo cows and buffalo cows that experienced embryo mortality after artificial insemination (AI). Values are means ± standard errors. Bars with different lowercase letters within each time are significantly different at *p* < 0.05.

**Figure 4:**
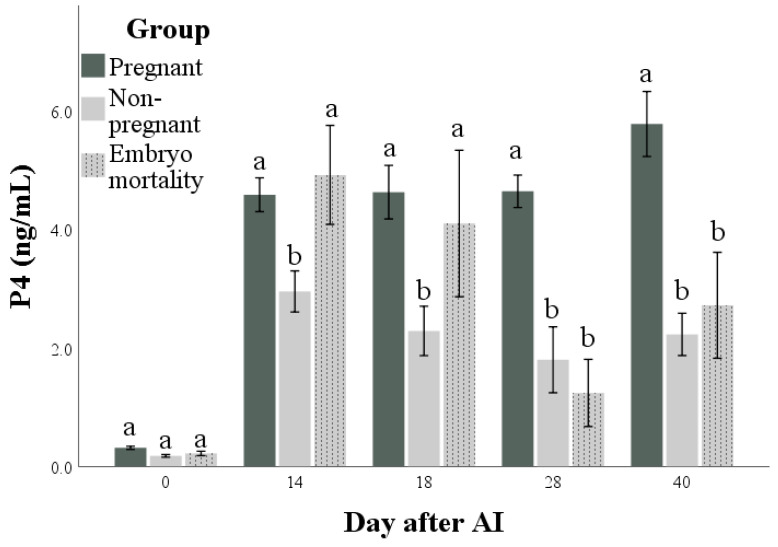
Progesterone (P4) concentrations in pregnant and non-pregnant buffalo cows and buffalo cows that experienced embryo mortality after artificial insemination (AI). Values are means ± standard errors. Bars with different lowercase letters within each time are significantly different at *p* < 0.05.

Both PAG and P4 concentrations (Figure 3 and Figure 4, respectively) were influenced by time, group, and their interactions (for all: *p* < 0.001). The highest marginal means of PAGs and P4 were found in the Pregnant group. Differences in the marginal means between the Non-pregnant and Embryo mortality groups were found for PAG (higher in the Embryo mortality group than in the Non-pregnant group, *p* < 0.001) but not for P4 concentrations. Multiple comparisons highlighted the differences between groups at each time point. In particular, they showed that there were no differences in P4 and PAG concentrations between the Pregnant and Embryo mortality groups until day 18 after AI. On the same days, the Non-pregnant group always had the lowest values. At day 28, the Embryo mortality group showed intermediate PAG concentrations, while the P4 concentrations did not differ from those of the Non-pregnant group. 

Table 1 shows the results of the regressions that included IFNt as the independent variable. They demonstrate that PAGs from day 18 (*p* < 0.01), and P4 from day 28 (*p* < 0.05) were positively associated with IFNt values. The highest amounts of explained variance (R^2^) were found for the models including PAGs as the dependent variable (more than 35% of total variation in PAGs could be accounted for by the variance of the IFNt).

Table 2 shows the indices of biological variation in the parameters from day 14 to 40. The lowest index of individuality (indicating a marked individuality) was found for IFNt (all <1), the highest for P4.

## 4. Discussion

To the best of our knowledge, this is the first report on IFNt protein detection in circulating maternal blood in buffalo.

Our study showed that from day 14 post AI it is possible to detect the protein in the blood. The mean IFNt concentrations in pregnant and non-pregnant buffalo cows showed significant differences at all sampling points. Our results are in agreement with the findings reported in bovines by [32,33]. These authors reported that the plasma IFNt was detectable from day 14 post AI, with higher values in pregnant cows compared to non-pregnant or late-embryonic-mortality cows. These data reflect the biological function of IFNt, which is involved in the mechanism of maternal recognition of pregnancy. It has been reported that IFNt is secreted by the trophoblast between days 12 and 26 of pregnancy in ruminants [3], buffalo included [7]. 

In our work, buffalo cows that experienced embryo mortality appeared to have lower values of circulating IFNt compared to pregnant animals from day 14 post AI, although the difference was not significant. The production of appropriate levels of IFNt is necessary for the survival of the embryo. Talukder et al. [45], in a study on cultured bovine endometrial explants, suggest that IFNt acts on the uterus in a dose- and time-dependent manner and that timely exposure of the endometrium to sufficient IFNt is essential for appropriate signalling that ensures successful pregnancy establishment. Kowalczyk et al. [3] reported that the process of IFNt significantly affected the embryo elongation process. Failures or delays in trophoblast elongation and/or embryonic development result in loss of pregnancy, possibly due to suboptimal histotroph [46,47].

The results obtained for IFNt expression showed no significant differences between the groups and time points considered, according to our previous study on buffalo [13]. Actually, studies in bovine reported that IFNt expression in the blood is not easily detectable [32,48], despite the fact that IFNt transits from the uterus into the systemic circulation to exert its effects on maternal physiology. Since it is difficult to evaluate the expression of IFNt in blood, the response of circulatory leucocytes to IFNt through the Interferon-Stimulated Genes (ISGs) was used as an alternative method for IFNt expression evaluation in ruminants [10,49,50]. In a previous work, we found that in buffalo, too, the ISGs expression proved to be a reliable peripheral biomarker for the prediction of pregnancy and embryo mortality during the peri-implantation period, with respect to IFNt expression. The ISGs were more strongly expressed in the pregnant than in the non-pregnant animals, in line with the observations reported by other authors [12,51,52].

As was to be expected, both PAG and P4 concentrations showed a higher value in pregnant animals than in non-pregnant animals [15,21,53]. The results of this study confirm PAG determination as a reliable method for pregnancy diagnosis and the follow up of trophoblastic function. The animals that experienced embryo mortality showed a decrease in PAG concentration starting from day 28 and reached the same value as the non-pregnant animals at day 40. These findings reflect those described in our previous work in buffalo [21,54] and those reported for bovine [55,56,57]. Regarding P4, although the concentration showed a significant difference between the pregnant and non-pregnant animals, it is important to highlight that the concentration of progesterone in the first weeks of pregnancy reflects the function of the CL more than the presence of an embryo [28,58]. The maintenance of P4 production by the CL is essential for embryo survival and successful pregnancy; IFNt, through the reduction in the oxytocin receptors in the endometrium, prevents the production of PGF2α and luteolysis, playing a pivotal role in the maternal recognition of pregnancy [1,8,46]. 

In addition to its numerous paracrine actions, the mode of IFNt transportation and endocrine actions still remains uncertain. The direct action of IFNt on the CL may confer the resistance of the CL to the luteolytic pulses of PGF2α; in addition, IFNt may control antiapoptotic mechanisms and cell survival genes to ensure luteal cell differentiation that prolongs the luteal life span [59]. Although attempts have been made in bovines to determine the presence of IFNt in body fluids using various assays, they were unsuccessful [34,60]. 

Comparing the IFNt values with those of PAGs or P4 through a regression analysis, we found that the values of IFNt were positively associated with those of PAGs starting from day 18 and those of P4 starting from day 28 post AI. The close association between IFNt and PAGs could be due to the fact that both are secreted by the mononuclear cells of the trophoblast around the peri-implantation period, and both are involved in the MRP: the IFNt exerts an antiluteolytic action by inhibiting PGF2α release, and PAGs exert a pro-luteotropic action by increasing PGE2 synthesis. Studies in vitro by Del Vecchio et al. [19,61] and Weems et al. [20,62] suggest that PAGs are a regulator of luteal prostaglandin secretion, and this, in turn, may help regulate P4 production. In fact, the administration of PAGs to cultured endometrium induced an increase in PGE2 in the media. The positive association between IFNt and P4 confirms the importance of IFNt in the maintenance of CL activity in overcoming the critical period of MRP, promoting the attachment of the embryo and the continuation of the pregnancy [32,63]. Kerbler et al. [63] observed a positive correlation between maternal plasma P4 and IFNt from the bovine conceptus by day 18 after a 24 h culture. In fact, IFNt produced from the trophectoderm results in the elongation of the conceptus, and the elongated or more voluminous embryos, in turn, produce more P4 to sustain the pregnancy [30]. Therefore, the positive associations in buffalo maternal circulation among IFNt, PAGs and P4 can reflect the presence of a viable embryo.

Despite the fact that circulating IFNt was detectable in blood with a significant difference between pregnant and non-pregnant buffalo cows, the analysis of biological variation showed the lowest index of individuality for IFNt, when compared to P4 or PAG. The great individual variability in IFNt was found both in the entire sample and in the individual groups of pregnant, non-pregnant animals and animals that experienced embryo mortality. This finding suggests that the detection of circulating IFNt, unfortunately, cannot be utilised as a reliable marker of pregnancy.

## 5. Conclusions

This is the first report that shows it is possible to detect the maternal circulation of IFNt in buffalo cows during the early period of gestation, showing different concentrations among pregnant animals, non-pregnant animals and animals that experience embryo mortality. The great individual variability in circulating IFNt makes this analysis unsuitable for early pregnancy diagnosis.

The close associations among IFNt, PAGs and P4 during the sensitive period in which the conceptus must manifest its presence demonstrate that all three molecules work together for fetal–placental well-being and support of pregnancy.

## Figures and Tables

**Table 1:** Results of the linear regression analyses that included IFNt as the predictor.

Dependent Variable	Day	Unstandardized Coefficients		*p* Value	R^2^
		B	Std. Error		
PAG *	14	0.093	0.063	0.158	0.10
	18	0.140	0.035	<0.001	0.46
	28	0.224	0.068	0.004	0.35
	40	0.725	0.189	0.001	0.44
P4	14	0.421	0.409	0.318	0.06
	18	0.728	0.451	0.122	0.12
	28	0.979	0.413	0.028	0.22
	40	1.020	0.433	0.029	0.22

* after Log(x + 1) transformation.

**Table 2:** Indices of biological variation of pregnancy-associated glycoproteins (PAG), progesterone (P4), and interferon (IFNt) from day 14 to 40, calculated for the entire sample (regardless of group) and stratified by group.

Parameter	Group	CVintra	CVinter	Index of Individuality (II)
PAG	Entire sample	94.3	127.0	0.742
	Pregnant	134.3	138.2	0.972
	Non-pregnant	70.1	66.2	1.059
	Embryo mortality	64.2	64.3	0.998
P4	Entire sample	52.5	42.2	1.242
	Pregnant	24.7	25.0	0.990
	Non-pregnant	70.8	65.4	1.083
	Embryo mortality	75.1	65.6	1.144
IFNt	Entire sample	27.8	34.2	0.812
	Pregnant	30.3	36.3	0.837
	Non-pregnant	20.9	23.8	0.881
	Embryo mortality	30.4	31.0	0.983

CVintra = Intra-individual coefficient of variability. CVinter = inter-individual coefficient of variability.

## Data Availability

The raw data supporting the conclusions of this article will be made available by the authors on request.
